# Dexmedetomidine regulates exosomal miR-29b-3p from macrophages and alleviates septic myocardial injury by promoting autophagy in cardiomyocytes via targeting glycogen synthase kinase 3β

**DOI:** 10.1093/burnst/tkae042

**Published:** 2024-11-04

**Authors:** Tianyi Yu, Hsinying Liu, Min Gao, Dan Liu, JiaQiang Wang, Jie Zhang, Jizhuang Wang, Peilang Yang, Xiong Zhang, Yan Liu

**Affiliations:** Department of Burn, Ruijin Hospital, Shanghai Jiao Tong University School of Medicine, 197 Ruijin Er Road, Shanghai 200025, China; Department of Burn, Ruijin Hospital, Shanghai Jiao Tong University School of Medicine, 197 Ruijin Er Road, Shanghai 200025, China; Department of Burn, Ruijin Hospital, Shanghai Jiao Tong University School of Medicine, 197 Ruijin Er Road, Shanghai 200025, China; Department of Burn, Ruijin Hospital, Shanghai Jiao Tong University School of Medicine, 197 Ruijin Er Road, Shanghai 200025, China; Department of Burn, Ruijin Hospital, Shanghai Jiao Tong University School of Medicine, 197 Ruijin Er Road, Shanghai 200025, China; Department of Burn, Ruijin Hospital, Shanghai Jiao Tong University School of Medicine, 197 Ruijin Er Road, Shanghai 200025, China; Department of Burn, Ruijin Hospital, Shanghai Jiao Tong University School of Medicine, 197 Ruijin Er Road, Shanghai 200025, China; Department of Burn, Ruijin Hospital, Shanghai Jiao Tong University School of Medicine, 197 Ruijin Er Road, Shanghai 200025, China; Department of Burn, Ruijin Hospital, Shanghai Jiao Tong University School of Medicine, 197 Ruijin Er Road, Shanghai 200025, China; Department of Burn, Ruijin Hospital, Shanghai Jiao Tong University School of Medicine, 197 Ruijin Er Road, Shanghai 200025, China

**Keywords:** Exosome, miR-29b-3p, Dexmedetomidine, Sepsis, Cardiomyocytes, Macrophages, Autophagy, Apoptosis, Glycogen synthase kinase 3β

## Abstract

**Background:**

Our previous research suggested that dexmedetomidine (Dex) promotes autophagy in cardiomyocytes, thus safeguarding them against apoptosis during sepsis. However, the underlying mechanisms of Dex-regulated autophagy have remained elusive. This study aimed to explore the role of exosomes and how they participate in Dex-induced cardioprotection in sepsis. The underlying microRNA (miRNA) mechanisms and possible therapeutic targets for septic myocardial injury were identified.

**Methods:**

We first collected plasma exosomes from rats with sepsis induced by caecal ligation and puncture (CLP) with or without Dex treatment, and then incubated them with H9c2 cells to observe the effect on cardiomyocytes. Subsequently, the differential expression of miRNAs in plasma exosomes from each group of rats was identified through miRNA sequencing. miR-29b-3p expression in circulating exosomes of septic or non-septic patients, as well as in lipopolysaccharide-induced macrophages after Dex treatment, was analysed by quantitative real-time polymerase chain reaction (qRT–PCR). The autophagy level of cardiomyocytes after macrophage-derived exosome treatment was assessed by an exosome tracing assay, western blotting, and an autophagic flux assay. Specific miRNA mimics and inhibitors or small interfering RNAs were used to predict and evaluate the function of candidate miRNA and its target genes by qRT-PCR, annexin V/propyl iodide staining, autophagy flux analysis, and western blotting.

**Results:**

We found that plasma-derived exosomes from Dex-treated rats promoted cardiomyocyte autophagy and exerted antiapoptotic effects. Additionally, they exhibited a high expression of miRNA, including miR-29b-3p. Conversely, a significant decrease in miR-29b-3p was observed in circulating exosomes from CLP rats, as well as in plasma exosomes from sepsis patients. Furthermore, Dex upregulated the lipopolysaccharide-induced decrease in miR-29b-3p expression in macrophage-derived exosomes. Exosomal miR-29b-3p from macrophages is thought to be transferred to cardiomyocytes, thus leading to the promotion of autophagy in cardiomyocytes. Database predictions, luciferase reporter assays, and small interfering RNA intervention confirmed that glycogen synthase kinase 3β (GSK-3β) is a target of miR-29b-3p. miR-29b-3p promotes cardiomyocyte autophagy by inhibiting GSK-3β expression and activation.

**Conclusions:**

These findings demonstrate that Dex attenuates sepsis-associated myocardial injury by modulating exosome-mediated macrophage–cardiomyocyte crosstalk and that the miR-29b-3p/GSK-3β signaling pathway represents a hopeful target for the treatment of septic myocardial injury.

Highlights

- Dexmedetomidine was recently found to exert myocardial protection by regulating exosome-mediated macrophage–cardiomyocyte crosstalk, thus providing more comprehensive pharmacological evidence for the use of dexmedetomidine in relieving septic myocardial injury.
- The expression of exosomal miR-29b-3p is significantly lower in the plasma of cases with sepsis and in rats subjected to a sepsis model. Thus, plasma miR-29b-3p can be considered a promising new candidate biomarker or therapeutic target for sepsis.
- The regulation of GSK-3b Ser9 phosphorylation is a new target and mechanism by which miR-29b-3p regulates autophagy.

## Background

Sepsis, defined as a complex pathophysiological process, has emerged as a significant contributor to persistently elevated mortality rates among critically ill patients [1]. Septic myocardial injury, a prevalent complication of sepsis, is closely linked to increased mortality in patients with sepsis [2]. Therefore, strategies aimed at protecting affected hearts from sepsis could reduce mortality.

Excessive infiltration of immune cells is a significant pathological feature of septic myocardial injury. In this context, macrophages act as the dominant immune cells, controlling the progression and resolution of disease [3]. The primary function of macrophages is to eliminate unwanted material through phagocytosis, and they are also known to have tissue-specific functions, which are important in the heart and serve as imperative regulators during cardiac repair processes [4].

Exosomes are bioactive substances that have been discovered in recent years and that can mediate cell-to-cell communication through their ‘paracrine’ effects [5]. These ‘paracrine’ effects involve the transfer of endogenous mRNAs and microRNAs (miRNAs) between cells via exosomes [6]. Microenvironmental stimulation results in epigenetic remodelling of cells, accompanied by corresponding changes in the composition of exosome contents (miRNA, mRNA, lipids, and proteins) secreted by cells [7]. Recent research has underscored the significance of exosome-mediated crosstalk between cardiosphere-derived cells and macrophages in maintaining cardiac homeostasis, with aberrant crosstalk potentially contributing to increased cardiac damage [8]. Furthermore, the paracrine effect of cells can be enhanced by various factors, including drugs, hormones, and physical factors. These factors regulate the biological behaviour and function of cells.

Sepsis-induced cardiomyocyte apoptosis has been identified as a critical factor in septic myocardial injury [9]. Recent studies in models of myocardial infarction, ischaemia–reperfusion, and chronic myocardial ischaemia have suggested that to maintain the normal function of the heart, the body responds to these conditions by increasing autophagosome formation, thus implicating activated autophagy as a protective mechanism [10–12]. Our previous *in vivo* study showed that dexmedetomidine (Dex) can mitigate septic heart injury by limiting inflammation and apoptosis through the regulation of autophagy [13]. However, the precise underlying mechanisms and targets by which Dex regulates autophagy remain obscure. The purpose of the present research was to investigate the effects and mechanisms underlying the ability of Dex-pretreated plasma-derived exosomes (Dex-Exos) to ameliorate septic heart injury. To gain insights into the role of Dex-induced exosomes and their involvement in septic heart injury, we employed RNA-seq to profile miRNAs in exosomes derived from the plasma of sepsis rats induced by caecal ligation and puncture (CLP) (CLP-Exos). To predict and evaluate the function of candidate miRNAs and their target genes, specific miRNA mimics and inhibitors or small interfering RNAs (siRNAs) were employed. This was achieved through quantitative real-time polymerase chain reaction (qRT-PCR), annexin V/propyl iodide staining, autophagy flux analysis, and western blotting.

In this study, we investigated whether Dex upregulated exosomal miR-29b-3p levels in macrophages, which correspondingly promoted cardiomyocyte autophagy. Moreover, the function of exosomal miR-29b-3p in septic cardiac injury and its mechanism were further explored. Our findings provide more comprehensive pharmacological evidence for the use of Dex in the treatment of myocardial injury in sepsis patients and may provide innovative strategies for its prevention and for the treatment of patients with septic cardiac injury.

## Methods

### Ethics approval

Blood samples collected from septic patients and intensive care unit (ICU) nonseptic patients were approved by the independent ethics committee of Shanghai Jiao Tong University School of Medicine and all of the participants provided informed consent.

Animals were procured from the Shanghai Laboratory Animal Center and were raised in the Animal Science Center of Ruijin Hospital, Shanghai Jiao Tong University School of Medicine (RJH). All of the animal procedures adhered to the guidelines established by the Animal Care Committee of RJH, and all of the animal experimental operations received approval from the Institutional Animal Care and Use Committee of RJH (RJ2023020).

### Human blood samples

Blood samples were collected from patients diagnosed with sepsis and from individuals serving as controls in the ICU. A total of 2–3 ml of blood was collected and subsequently subjected to plasma extraction, and was then stored at −80°C in order to facilitate subsequent exosome extraction [14]. The patients diagnosed with sepsis were aged between 20 and 60 years and were admitted to the ICU of Ruijin Hospital. Plasma quantification of miR-29b-3p was performed in patients with sepsis (*n* = 8) during the first 3 days following ICU admission and compared with that in ICU nonseptic patients (nonsepsis controls, *n* = 8).

### CLP-induced experimental sepsis

Male Wistar rats, aged 7–8 weeks and weighing 200 ± 15 g, were randomly allocated to one of three groups: the sham group (*n* = 9), CLP group (*n* = 9), and CLP + Dex group (*n* = 9). The CLP model was employed to induce polymicrobial sepsis [15]. The rats were administered an intraperitoneal injection of 1% phenobarbital (40 mg/kg body weight), disinfected for the surgical procedure, and the operation was conducted via a 1.5-cm incision in the midline of the rat abdomen under qualified anaesthesia. The caecum is located and then perforated with a needle to release feces into the abdominal cavity. There was no caecal perforation in the sham group, and the incision was sutured after laparotomy. Rats were injected subcutaneously with bupivacaine and buprenorphine to relieve postoperative labour pain. All rats were intraperitoneally injected with lactate Ringer’s fluid (30 ml/kg body weight) after injury for liquid resuscitation and then fed alone. The rats in the Dex treatment group were injected with Dex (50 μg/kg) 3 h after CLP, while the animals in the other groups received an equal volume of 0.9% NaCl. Following the injection, the rats were returned to their individual cages.

### Cell treatment

#### Rat and human cardiomyocyte cell lines

The rat cardiomyocyte cell line H9c2 (#CRL-1446) and the human cardiomyocyte cell line AC16 (#BNCC339980) were procured from ATCC and BNCC, respectively. These cell lines were cultured according to a standard protocol. For the autophagy induction assay, H9c2 cells and AC16 cells were treated with plasma-derived or macrophage-derived exosomes (10 μg/ml) for 24 h.

#### THP-1 monocyte differentiation and macrophage culture

THP-1 monocytes (human monocytic cell line ATCC#TIB-202) were treated with 50 ng/ml phorbol 12-myristate 13-acetate for 48 h to induce differentiation into M0 macrophages by Day 3. THP-1 macrophages were cultured in a standard protocol. Once the THP-1 cells had reached a confluence of 80–90%, they were incubated with RPMI-1640, which had been processed to remove the exosomes from foetal bovine serum by ultracentrifugation at 100 000 × g overnight. Additionally, the cells were pretreated with or without Dex for 1 h and then treated with lipopolysaccharide (LPS) for 24 h at 37°C with 5% CO_2_. Afterwards, the supernatants were collected for exosome isolation. The reagents that were used in this study are listed in supplementary Table 1 (see online supplementary material).

### Exosome isolation and identification

#### Isolation of exosomes

Exosomes were isolated for cell culture following a previously established method [16]. Briefly, the collected plasma and THP-1 cell culture solution were first separated through centrifugation at 3000 rpm for 30 min. Subsequently, the collected supernatant was further centrifuged at 13 000 rpm for 30 minutes. The final supernatant was passed through a 0.1 μm membrane filter (Merck Millipore) and centrifuged at 36 000 rpm at 4°C for 2 h, employing a Ti-45 rotor. To prevent secreted protein contamination, the exosome particles were first re-suspended with DMEM and then washed with phosphate buffered saline (PBS) to obtain purified exosomes. Micro-BCA assay (Pierce, Rockford) was used to determine exosome concentration.

#### Exosome characterization

The isolated exosomes were subsequently fixed with 4% glutaraldehyde solution and placed onto a copper grid. The sections were then stained with 2% phosphotungstic acid for 30 s. The morphology of exosomes was observed using a transmission electron microscope (Tecnai G2 spititi FEI). Nanoparticle tracking analysis (NTA) was conducted using a NanoSight NS300 instrument (Malvern) to discern the size distribution of the particles. The expression of exosome markers, including cluster of differentiation antigen 63 (CD63) and alg-2-interacting protein x (Alix), as well as the negative marker β-actin, was detected via western blotting.

### Rat peripheral blood mononuclear cell isolation

For the extraction of rat peripheral blood mononuclear cells to serve as a negative control, the following protocol was used [17]. Blood specimens (5 ml) were collected from the rats into sodium heparin blood-collection tubes and diluted with RPMI-1640. The diluted blood was then transferred into 50-ml conical centrifuge tubes containing 15 ml of Ficoll-Paque reagent, followed by centrifugation at 500 × g for ~30 min at 20°C without braking. After centrifugation, the enriched cell fraction was transferred to another conical centrifuge tube for cell washing, RBC lysis, and protein extraction.

### Exosome miRNA sequencing and bioinformatics analysis

TRIzol was used to extract total RNA from exosome samples, and reverse transcription and PCR were performed. The quantity of total RNA was determined by means of a NanoDrop ND-2000 spectrophotometer (Thermo Scientific). The samples were labelled, hybridized on a microarray, and washed in accordance with the manufacturer’s standard protocols. The labelled and purified RNA was hybridized onto a microarray. Subsequently, scanning detection was performed with an Agilent G2505C scanner (Agilent Technologies). Differentially expressed miRNAs were identified by fold-change value and *P*-value calculated by t-test.

Bioinformatics analyses of the differentially expressed exosomal miRNAs that were identified in this study were conducted using OmicStudio tools, which are accessible at https://www.omicstudio.cn/home. A heatmap was generated using the Advanced Heatmap Plots tool to provide a visual representation of all differentially expressed miRNAs.

### Detection of exosome uptake by cardiomyocytes

Exosomes extracted from the supernatants of macrophages were labelled with PKH67 red fluorescence (Umibio, UR52302) following the experimental procedures outlined in the protocol. Briefly, 200 μl of exosomes was mixed with 45 μl of Diluent C and 5 μl of PKH67 dye, and the mixture was incubated for 10 min at 25°C. PKH67-labelled exosomes were subsequently collected via ultracentrifugation at 100 000 × g for 70 min at 4°C.

Exosomes labelled with PKH67 or PKH67-PBS control were incubated separately with AC16 cells cultured on confocal dishes for 12 h. The cytoskeleton of AC16 cells was stained with 100 nM FITC-phalloidin (Yeasen) for 30 min at 25°C. The nuclei of AC16 cells were stained with 20 mM 4′,6-diamidino-2′-phenylindole solution (Thermo Fisher) for 10 min at room temperature. The cells were observed under a Zeiss LSM 880 confocal microscope (Zeiss, Wetzlar, Germany).

### RNA extraction and qRT–PCR

Total RNA from both exosomes and cells was isolated using TRIzol (Thermo Fisher). mRNA was reverse-transcribed into cDNA using a HiScript III RT Reagent Kit (Vazyme). For quantitative RT-PCR of miRNA, an miRNA 1st Strand cDNA Synthesis Kit (Vazyme) was applied, and stem–loop structure was utilized to facilitate the reverse transcription process. A real-time PCR system (Applied Biosystems 7500, USA) was used for PCR reaction detection. β-Actin and U6 were used to assess mRNA and miRNA levels in samples, respectively. The relative expression levels of mRNA or miRNA were calculated using the 2^−ΔΔCT^ method. The primers that were used in this study are listed in supplementary Table 1 (see online supplementary material).

### Analysis of autophagic flux

The autophagy indicator stubRFP-sensGFP-LC3 lentivirus was obtained from Genomeditech (Shanghai, China). Briefly, H9c2 cells and AC16 cells were infected with stubRFP-sensGFP-LC3 lentivirus for 24 h. Afterwards, puromycin at the appropriate concentration was added to screen for cells with stable expression. These cells were then seeded in a confocal dish at a density of 1 × 10^5^/dish. The expression of GFP and mRFP was visualized using a Zeiss LSM 880 confocal microscope (Zeiss, Wetzlar, Germany). Images were acquired using Zeiss Zen software.

### miR-29b-3p and si-glycogen synthase kinase 3β transfection *in vitro*

miR-29b-3p mimics and their negative control (miR-NC) were transfected into H9c2 and AC16 cells, and glycogen synthase kinase 3β (GSK-3β) siRNA (si-GSK-3β) along with its negative control (si-NC) were transfected into H9c2 and AC16 cells using Lipofectamine 2000 (Invitrogen, Carlsbad, CA, USA).

### Dual-luciferase reporter assay

The potential target of miR-29b-3p was predicted by utilizing the miRDB (mirdb.org), TargetScan (targetscan.org), and miRanda (microRNA.org) databases. Luciferase activities were analysed in H9c2 cells 24 h post-transfection using a dual-luciferase reporter assay system (Promega).

### Flow cytometry

The apoptosis rate was analysed by flow cytometry. Cell precipitation was obtained by centrifugation at 2100 rpm for 10 min. The cells were then re-suspended with 500 μl of PBS, and 10 μl of 500 μg/l RNase and 10 μl of 40 μg/l propyl iodide were added to the cell suspension, respectively. The suspension was incubated at room temperature for 10 min. The fluorescence uptake of propyl iodide by cells was measured by flow cytometry. Each experiment included at least 10 000 gated events, which were subsequently analysed using FlowJO 7.6.1 software.

### Western blot analysis

Protein samples were extracted from both cells and exosomes using specific procedures [14]. Equal quantities of protein were loaded and separated via SDS-PAGE. The target protein was detected by the use of peroxidase-conjugated secondary antibodies and enhanced chemiluminescence. The primary antibodies and reagents used in the experiments and their dilution are detailed in supplementary Table 2 and 3, see online supplementary material.

### Statistical analysis

The normality of the data distribution was tested using the Shapiro–Wilk test. Data that exhibited a normal distribution are expressed as the mean ± SD. Comparisons between two groups were performed using the two-tailed Student’s t-test. Multiple group comparisons were conducted via one-way ANOVA followed by Tukey’s multiple comparisons test with GraphPad Prism 9 software, as appropriate. A *P*-value < 0.05 was considered to indicate statistical significance.

## Results

### Plasma-derived exosomes from CLP rats with or without Dex treatment regulate apoptosis- and autophagy-related proteins in H9c2 cells

**Figure 1:**
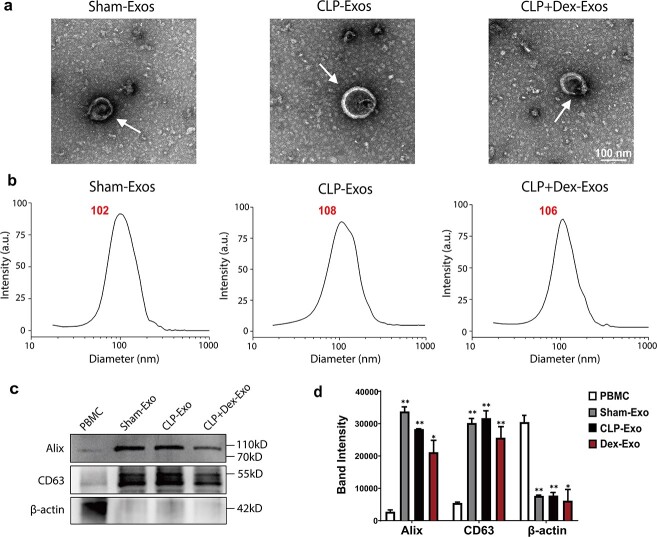
Characterization of rat plasma-derived exosomes. (**a**) Representative TEM images of plasma exosomes from rats in each group; scale bar:  100 nm. (**b**) Size distribution of plasma exosomes in each group, as detected using NTA. (**c**) Western blot analysis of the levels of the exosomal markers CD63 and Alix and the negative marker β-actin in plasma exosomes. Total protein from rat PBMCs was used as a negative control. (**d**) Quantitative analysis of Alix, CD63, and β-actin expression in plasma exosomes and PBMCs (*n* = 3). All of the experiments were independently performed three times. ^*^p < 0.05, ^**^p < 0.01 compared with PBMCs by one-way ANOVA followed by Tukey’s test. *Exos* exosomes, *Dex* dexmedetomidine

To investigate the effects of plasma-derived exosomes on cardiomyocytes, we isolated exosomes from the plasma of rats in the three groups: three from the sham group, three from the CLP group, and three from the Dex group. The morphological characteristics of the isolated exosomes were visualized using transmission electron microscopy (TEM) (Figure 1a). To provide a detailed view of the purified particles, we performed NTA to measure the size distribution of the vesicles. The diameters of nearly all of the particles were found to range from 30 to 150 nm, with an average size of 105.5 ± 6.1 nm (Figure 1b). Furthermore, in comparison to rat peripheral blood mononuclear cell lysates analysed via immunoblotting, the preparations of plasma exosomes exhibited a high enrichment of the exosomal marker proteins Alix and CD63, along with low expression of the negative marker β-actin (Figure 1c and d).

Autophagy is a crucial process in maintaining cellular homeostasis. Under autophagy activation, microtubule-associated-protein light-chain-3 (LC3)-I undergoes lipidation and is converted into LC3-II. The ratio of LC3-II/LC3-I is widely used as a marker of autophagy activation [18]. Beclin1, which is encoded by autophagy-related 6 (Atg6), is essential for initiating autophagosome formation during autophagy [19,20]. p62 is a ubiquitin-binding protein whose levels decrease when autophagy is induced and accumulate when autophagy is inhibited, thus p62 can be used as a marker to assess autophagy flux [20].

**Figure 2:**
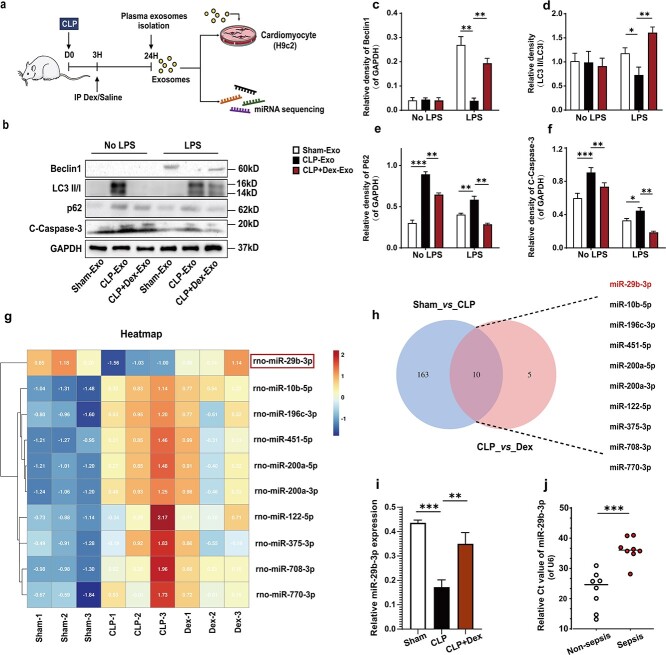
CLP + Dex-Exos induced rat cardiomyocyte autophagy and were rich in miR-29b-3p. (**a**) Schematic diagram of the experimental design used to establish the animal model and coculture. (**b–f**) Effect of CLP + Dex-Exos on the levels of autophagy-related proteins, as measured by western blotting. GAPDH was used as the loading control. The data are expressed as mean ± SD. (**g**) Heatmap of plasma exosomal miRNA sequencing data from rats in each group (*n* = 3). (**h**) Venn diagram depicting overlap between differentially expressed genes in the sham *vs* CLP (blue) and CLP *vs* CLP + Dex (red) pairwise comparisons of differentially expressed exosomal miRNAs according to our RNA sequencing results. (**i**) qRT–PCR analysis of miR-29b-3p expression in plasma exosomes from sham, CLP and CLP + Dex rats. (**j**) Expression of miR-29b-3p in plasma exosomes from patients with or without sepsis**.**  ^*^p < 0.05, ^**^p < 0.01, ^***^p < 0.005. *Cleaved-Caspase-3* cleaved cysteine–aspartic acid protease 3, *LC3A/B* microtubule-associated-protein light-chain-3 A/B, *P62* sequestosome 1, *GAPDH* glyceraldehyde-3-phosphate dehydrogenase, *SD* standard deviation, *Exos* exosomes, *Dex* dexmedetomidine, *CLP* cecal ligation and puncture

After LPS stimulation, treatment with CLP-Exos significantly decreased the expression of Beclin1 and the conversion of LC3 (LC3-I to LC3-II), while increasing the levels of p62 and the apoptotic protein cleaved caspase-3. In contrast, Dex-Exo treatment significantly upregulated LC3-II/LC3-I and Beclin1 expression but downregulated p62 and cleaved caspase-3 expression in H9c2 cells (Figure 2b–f). These findings suggest that Dex-Exos promote autophagy in H9c2 cardiomyocytes suppressed by CLP-Exos.

### miR-29b-3p expression is downregulated in CLP-Exos and upregulated in Dex-Exos

Due to the fact that miRNAs are essential inter-cellular signalling molecules enclosed within exosomes, we hypothesized that they are potentially associated with Dex-Exos-induced autophagy in H9c2 cells. It should be noted that the composition of exosomes is not random; rather, it is regulated by the cell source and environmental stressors [21,22]. To elucidate how Dex contributes to cardioprotection and induces autophagy, total RNA sequencing (RNA-seq) was performed on exosomes isolated from plasma of rats in the sham group (Sham-Exos), CLP rats (CLP-Exos) and Dex-treated CLP rats (Dex-Exos). These exosomal RNA sequences were then aligned to a database containing annotations for both short and long RNAs.

A total of 173 miRNAs exhibited significantly differential expression between the CLP-Exos and Sham-Exos groups, while 15 miRNAs demonstrated differential expression between the CLP-Exos and Dex-Exos groups (Figure 2h). Among the 10 miRNAs that were differentially expressed in both the CLP-Exos and Dex-Exos groups, miR-29b-3p was downregulated in the CLP-Exos group, whereas it was upregulated in the Dex-Exos group (Figure 2g).

To validate these results, we assessed miR-29b-3p expression in plasma-derived exosomes from patients with or without sepsis and CLP-induced sepsis rats treated with or without Dex by using a conventional real-time qPCR assay. According to the qRT–PCR results, miR-29b-3p was significantly downregulated in plasma-derived exosomes from sepsis patients compared to those from nonsepsis patients (Figure 2j). A summary of the clinical characteristics and demographics of the subjects is presented in supplementary Table 4 (see online supplementary material). Conversely, miR-29b-3p was greatly upregulated in exosomes from Dex-treated rats compared to those from CLP rats (Figure 2i). These findings suggest that miR-29b-3p is highly expressed in plasma exosomes derived from CLP rats after Dex intervention, thus indicating that exosomal miR-29b-3p may serve as a crucial target for promoting cardiomyocyte autophagy and reducing sepsis-induced cardiomyocyte apoptosis.

### Macrophage-derived exosomal miR-29b-3p regulates autophagy response and related protein expression in AC16 cardiomyocytes

Macrophages, as pivotal immune-regulating cells, play a crucial role in maintaining cardiomyocytes homeostasis during sepsis [23,24]. To investigate whether macrophages are involved in Dex-induced cardiomyocyte autophagy, we further verified that Dex stimulates the release of exosomal miR-29b-3p from macrophages and promotes cardiomyocyte autophagy.

**Figure 3:**
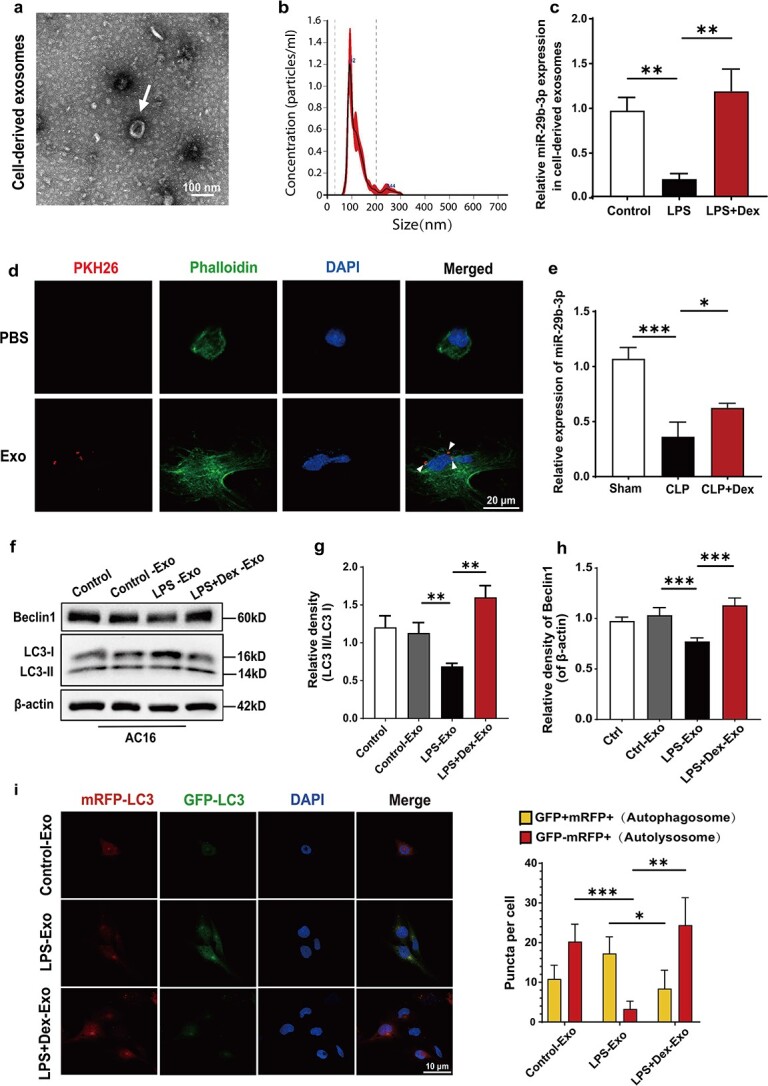
DEX upregulates macrophage-derived exosomal miR-29-3p and is transferred to cardiomyocytes to promote autophagy. (**a**) Representative TEM images of THP-1-derived exosomes; scale bar:  100 nm. (**b**) Size distribution of the THP-1-derived exosomes detected by NTA. (**c**) qRT–PCR analysis of the expression of exosomal miR-29b-3p in THP-1 macrophages in the PBS-, LPS-, and LPS + Dex-treated groups. The data are presented as means ± SD from three independent experiments. (**d**) Uptake of THP-1 macrophage-derived exosomes by AC16 cardiomyocytes was detected using confocal microscopy. Scale bar: 20 μm. (**e**) miR-29b-3p expression in heart tissues of rats. (**f–h**) Beclin1 and LC3II/I expression levels were evaluated using western blot analysis. β-Actin was used as the loading control. The data are expressed as mean ± SD. (**i**) Representative fluorescence images of AC16 cells expressing mRFP-GFP-LC3 and treated with control, LPS or LPS + Dex exosomes from THP-1 macrophages for 24 h. Nuclei were stained with DAPI. The numbers of autophagosomes (red/green double-coloured) and autolysosomes (red) in each cell were quantified (*n* = 10). Autophagic flux was evaluated by the change in the colour of GFP/mRFP. Scale bar:  10 μm. ^*^P < 0.05, ^**^P < 0.01, ^***^P < 0.005*. DAPI* 4′,6-Diamidino-2′-phenylindole, *CLP* cecal ligation and puncture, *LPS* lipopolysaccharides, *PBS* phosphate-buffered saline, *NTA* nanoparticle tracking analysis, *GFP* green fluorescent proteins, *Dex* dexmedetomidine

THP-1 monocytes were stimulated with phorbol 12-myristate 13-acetate and differentiated into M0 macrophages, which serve as a typical macrophage line for the assessment of intercellular communication [25]. We investigated the autophagic regulatory effect of these macrophages on cardiomyocytes by using exosomes derived from THP-1 macrophages. The morphological characteristics of the isolated exosomes were visualized using TEM (Figure 3a). Additionally, NTA was conducted to measure the size distribution of these vesicles (Figure 3b).

qRT–PCR was performed to measure the levels of miR-29b–3p in macrophage-derived exosomes treated with LPS (LPS-Exos) or LPS + Dex (LPS + Dex-Exos). Compared with that in the Ctrl-Exos (treated with PBS) group, the miR-29b-3p level in the LPS-Exos group was reduced, whereas it was upregulated in the LPS + Dex-Exos group (Figure 3c). The internalization of exosomes by target cells is a prerequisite for subsequent RNA transfer. To determine whether macrophage-derived exosomes can be internalized by cardiomyocytes, PKH67-labelled exosomes were incubated with AC16 cells for 12 h. Subsequent confocal microscopy imaging demonstrated that the PKH26-labelled exosomes (marked by the triangle indicators) adhered to the surface of the phalloidin-labelled AC16 cells and entered the cytoplasm, thus suggesting that the THP-1 macrophage-derived exosomes could be transferred to the cytoplasm of the AC16 cells (Figure 3d). Additionally, the expression of miR-29b-3p in heart tissues was significantly lower in CLP rats than in control rats and was partially increased after Dex treatment (Figure 3e).

To explore the impact of LPS + Dex-Exos on cardiomyocytes homeostasis, AC16 cells were treated with these exosomes. LPS-Exos inhibited autophagy by decreasing the level of Beclin1 and the ratio of LC3-II/LC3-I. Conversely, LPS + Dex-Exos treatment reversed the effects induced by LPS-Exos in AC16 cells (Figure 3f–h). Subsequently, we used the mRFP-GFP-LC3 assay to assess autophagic flux in AC16 cells to quantify autophagy activity. As the autophagolysosome is formed, the GFP signal is quenched, and a red fluorescence is observed. In cases where autophagosomes did not fuse with lysosomes, yellow dots were observed. Therefore, a high yellow dot/red dot ratio indicates a blocked autophagic process. The results of this assay demonstrated greater autophagosome–lysosome fusion in LPS + Dex-Exos-treated cells than in LPS-Exos-treated cells (Figure 3i). These findings suggest that Dex-induced macrophage-derived exosomes may facilitate autophagy in cardiomyocytes by transferring miR-29b-3p.

### Overexpression of miR-29b-3p reverses cardiomyocyte apoptosis and promotes autophagy suppressed by LPS *in vitro*

**Figure 4:**
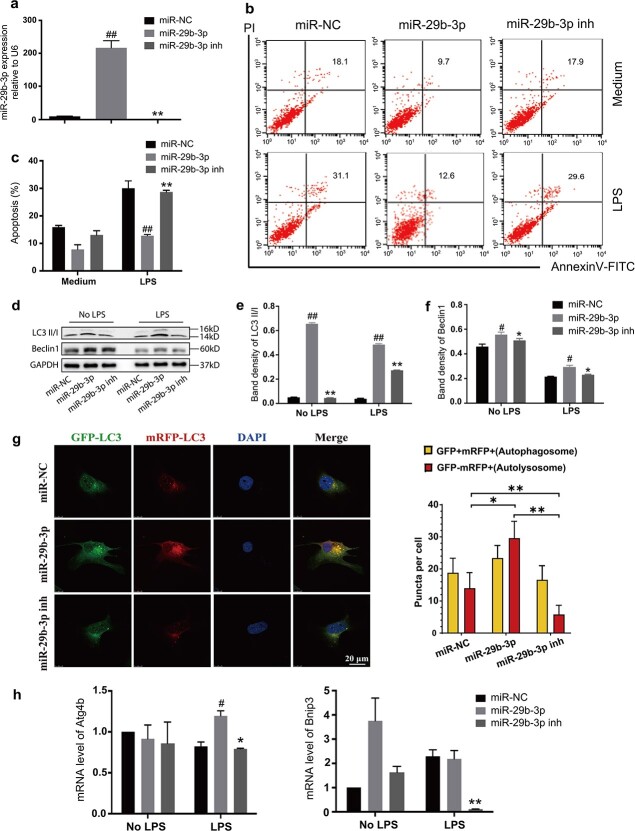
Overexpression of miR-29b-3p promoted autophagy and reduced LPS-induced apoptosis in H9c2 cells. (**a**) H9c2 cells were infected with miR-29b-3p mimics, miR-29b-3p-inh or NC lentivirus. The expression of miR-29b-3p in each group was determined using qRT–PCR. (**b**, **c**) H9c2 cells expressing miR-29b-3p mimics, miR-29b-3p-inh or NC were incubated without (upper panel) or with (lower panel) LPS for 24 h, and the cells were stained with PE-labelled Annexin V and 7-AAD to determine cellular apoptosis via flow cytometry. (**d–f**) Western blotting was used to measure the expression of autophagy-related proteins (Beclin1 and LC3BII/I) in H9c2 cells after the up- and down-regulation of miR-29b-3p. (**g**) Autophagic flux in each group of H9c2 cells. Scale bar: 100 μm. (**h**) qRT–PCR was used to measure autophagy-related gene expression in each group of H9c2 cells after the up- or down-regulation of miR-29b-3p. (^*^p < 0.05, ^##^*p* < 0.01 *vs* the miR-29b-3p group; ^*^p < 0.05, ^*^p < 0.01 *vs* the miR-29b-3p-inh group). *NC* negative control, *7-AAD* 7-aminoactinomycin D, *PI* propidium iodide, *GAPDH* glyceraldehyde-3-phosphate dehydrogenase, *LC3* microtubule-associated-protein light-chain-3, *LPS* lipopolysaccharides

To further confirm the functional efficacy of exosomal miR-29b-3p, we overexpressed miR-29b-3p in cardiomyocytes. We investigated whether miR-29b-3p could modulate LPS-induced cardiomyocyte apoptosis *in vitro*. The miR-29b-3p level was considerably greater in cardiomyocytes after transfection with miR-29b-3p mimics (Figure 4a and Figure S1a, see online supplementary material). There was no significant difference in the percentage of apoptosis among all groups at the basal level. However, in cells treated with LPS, the percentage of apoptotic cardiomyocytes infected with miR-29b-3p mimics was considerably lower than that in cells transfected with the negative group. The apoptosis rate in cells transfected with miR-29b-3p inhibitor was much greater than that in cells transfected with the mimics (Figure 4b and c and Figure S1b and c).

**Figure 5:**
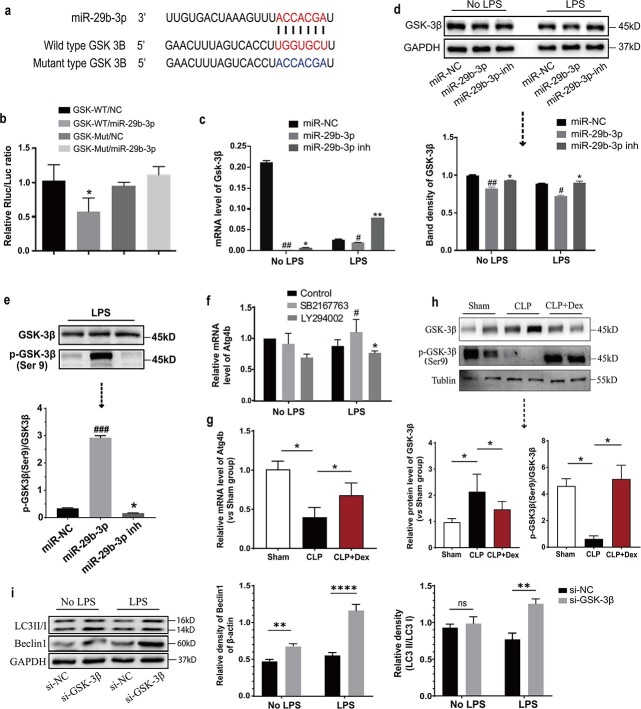
miR-29b-3p targets GSK-3β and inhibits its expression and phosphorylation, thus inducing autophagy in cardiomyocytes. (**a**) The target region of miR-29b-3p in the GSK-3β 3′-UTR was determined and a corresponding mutant was designed. (**b**) Wild-type or mutant GSK-3β 3′-UTR reporter vector was cotransfected into H9c2 cells with miR-29b-3p or miR-29b-3p-inh, and luciferase activity was assayed 48 h after transfection. (**c**, **d**) Relative transcriptional and protein expression levels of GSK-3β were assessed using qRT–PCR and western blotting. (**e**) Western blotting was used to measure the phosphorylation level of GSK-3β at Ser9 in each group after the up- or down-regulation of miR-29b-3p. (**f**) Expression of the autophagy-related gene Atg4 was measured using qRT–PCR in H9c2 cells after treatment with the GSK-3β inhibitor SB216763 (10 μM) or the PI3K inhibitor LY294002 (50 μM) for 24 h. (**g**) mRNA level of Atg4b in heart tissues of rats. (**h**) Western blot analysis of GSK-3β and phospho-GSK-3β (Ser9) expression in the heart tissue of rats in each group. The blots are representative of at least three independent experiments with similar results. (**i**) Western blotting was used to measure the expression of autophagy-related proteins Beclin1 and LC3BII/I in H9c2 cells after the knockdown of GSK-3β. ^#^*p* < 0.05, ^##^*p* < 0.01, ^###^*p*< 0.005 *vs* the miR-NC or control group; ^ns^*p* > 0.05,^*^p < 0.05, ^**^p < 0.01, ^****^p < 0.001 *vs* the miR-29b-3p-inh group; *ns* not significant, *LPS* lipopolysaccharides, *CLP* cecal ligation and puncture, *GSK-3β* glycogen synthase kinase 3β

Western blot analysis (Figure 4d–f and Figure S1d) demonstrated that the conversion ratio of LC3 and the level of Beclin1 were elevated in cardiomyocytes transfected with miR-29b-3p mimics in comparison to those in cells transfected with the negative control. The miR-29b-3p-inh inhibitor decreased the LC3 conversion and the level of Beclin1. The mRFP-GFP-LC3 assay showed yellow dots (representing autophagosomes) and red dots (representing autophagosomes–lysosomes fusion). An increase in the number of red dots and the ratio of red/yellow dots indicate the activation of autophagy. As shown in Figure 4g and Figure S1e, the miR-29b-3p mimic increased the number of red dots and the red/yellow dot ratio. Cells transfected with the negative control and miR-29b-3p inhibitor showed almost yellow diffusion and little red signal. The qRT–PCR results also confirmed that the transcriptional level of the autophagy-related gene Atg4b can be increased by transfection of cardiomyocytes with miR-29b-3p mimics, whereas the mRNA expression levels of other autophagy genes (Bnip3) were reduced in the miR-29b-3p-inhibitor group (Figure 4h).

In summary, these data demonstrated that in both human- and rat-derived cardiomyocytes, miR-29b-3p is essential for alleviating LPS-induced autophagy blockade and apoptosis in cardiomyocytes.

### miR-29b-3p promotes autophagy by affecting GSK-3β activation

To explore the role of miR-29b-3p in promoting autophagy in cardiomyocytes, we utilized three bioinformatics databases (miRDB, TargetScan, and miRanda) to find its potential candidate target genes. The 3′-UTR of miR-29b-3p and GSK-3β was observed to be completely complementary, suggesting that miR-29b-3p may play a regulatory role in GSK-3β activation (Figure 5a). Compared to that in the cotransfected control and reporter vector groups, the activity of firefly luciferase in the mimic group was found to be significantly lower (Figure 5b), thus suggesting that GSK-3β is a direct target gene of miR-29b-3p. As the seven seed sequences (TGGTGCT) on the GSK-3β 3′-UTR predicted by the software were deleted, inhibition of the firefly luciferase reporter gene by miR-29b-3p disappeared (Figure 5b). Both qRT-PCR and Western blot analyses results showed down-regulation of GSK-3β expression in H9c2 cells transfected with miR-29b-3p mimics (Figure 5c and d).

**Figure 6:**
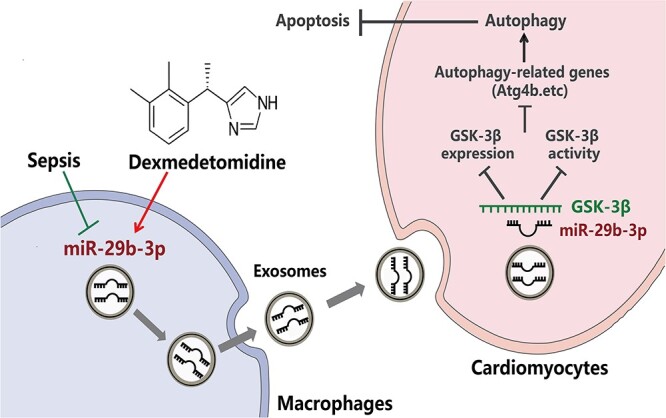
Schematic diagram showing how Dex regulates the transfer of macrophage-derived exosomal miR-29b-3p to cardiomyocytes and alleviates sepsis-induced cardiomyopathy by promoting autophagy and reducing apoptosis in cardiomyocytes by targeting GSK-3β

GSK-3β is a multifunctional protein kinase that plays a pivotal role in regulating cellular behaviours, including cell metabolism, differentiation, and proliferation [26,27]. GSK-3β is typically considered to be constitutively activated by autophosphorylation at Tyr216 and inactivated by phosphorylation at Ser9. The activity of GSK-3β has previously been evaluated by its ability to inhibit Ser9 phosphorylation [28–30]. In our study, we found that compared to cardiomyocytes treated with Sham-Exos, CLP-Exos downregulated GSK-3β Ser9 phosphorylation, whereas Dex-Exos upregulated this phosphorylation (supplementary Figure S2a, see online supplementary material). Furthermore, overexpression of miR-29b-3p was found to significantly elevate the phosphorylation of GSK-3β at Ser9 in cardiomyocytes treated with LPS.

We treated H9c2 cells with the GSK-3β inhibitor SB216763 and the agonist LY294002. We observed that the autophagy-related gene Atg4b considerably increased in the GSK-3β inhibitor group and significantly decreased in the agonist group, thus indicating that the phosphorylation of GSK-3β regulates the autophagy-related gene Atg4b (Figure 5f). The mRNA level of Atg4b and the expression and activation of GSK-3β in rat heart tissue also exhibited the same trend (Figure 5g and h).

Consistent with these findings, the blockade of GSK-3β expression reversed cardiomyocyte apoptosis (Figure S2b) and upregulated Beclin1 expression and the LC3 II/I ratio (Figure 5i and Figure S2c), thus suggesting that the knockdown of GSK-3β activated autophagy.

In summary, these data collectively demonstrate that exosomal miR-29b-3p promotes autophagy to protect cardiomyocytes by directly targeting GSK-3β and inhibiting its expression and activation (Figure 6).

## Discussion

In this study, our primary focus was to elucidate the mechanism underlying Dex-mediated cardioprotection in sepsis. The principal conclusions can be summarized as follows: (i) Dex-Exos can enhance autophagy and mitigate apoptosis downregulated by LPS induction; (ii) miR-29b-3p is downregulated in the CLP-Exos group compared to the Sham-Exos group but upregulated in the Dex-Exos group; (iii) Dex can regulate macrophage–cardiomyocyte crosstalk and induce the release of macrophage-derived exosomal miR-29b-3p, which acts on cardiomyocytes to promote autophagy; (iv) overexpression of miR-29b-3p can induce autophagy and reduce cardiomyocyte apoptosis; and (v) miR-29b-3p directly targets GSK-3β and inhibits GSK-3β activation, thus consequently enhancing Atg4b and promoting autophagy. Our study provides valuable insights as it is the first study to demonstrate that during sepsis Dex induces macrophages to deliver miR-29b-3p-enriched exosomes to cardiomyocytes. This mechanism plays a pivotal role in promoting cardiomyocyte autophagy and may serve as a crucial factor in how Dex exerts its cardioprotective effects. These results contribute to a more comprehensive understanding of the cardioprotective benefits of Dex during sepsis, thus providing more information on potential therapeutic avenues. Further research and clinical investigations in this area could lead to improved strategies for sepsis management.

The pathological manifestations of septic myocardial injury were excessive inflammatory cell infiltration, interstitial fibrosis, and mitochondrial injury. [31]. Disruption of mitochondrial homeostasis by inflammatory mediators, oxidative stress, and nitric oxide enhance apoptosis [32–34]. The intricate balance between mitochondrial stress, autophagy signalling, and mitochondrial biogenesis is essential for cardiomyocyte homeostasis [35–37]. It has been reported that the administration of Dex has beneficial effects on sepsis by stabilizing the microcirculation and mitigating the sympathetic impact on ventricular repolarization [38,39]. Our previous study demonstrated that Dex attenuated pathological changes in the myocardium of sepsis rats by inducing an autophagy response. The inhibition of autophagy reversed the protective effects of Dex, including reductions in apoptosis and inflammation [13]. Nevertheless, the underlying mechanisms and targets of Dex are incompletely understood, and some studies have suggested that Dex can aggravate LPS-induced myocardial dysfunction [40]. Therefore, further explorations of more precise mechanisms and targets of Dex may lead to the discovery of promising therapeutic strategies for treating septic myocardial injury and preventing adverse drug reactions. Studies have suggested that the *in vivo* benefits of Dex may be attributed to its interactions with circulating and tissue-resident macrophages, thus resulting in the reduced release of proinflammatory mediators by macrophages [41,42].

The release of various bioactive mediators into the bloodstream upon endotoxin stimulation plays a significant role in the onset and progression of sepsis [43]. Among these components, exosomes, which are a category of nanovesicles actively released from cells, play a pivotal role in influencing the function of recipient cells by delivering information through this abundant component [44]. Moreover, in response to various stimuli, such as toxins, hypoxia, and certain pathological conditions, changes in the composition of exosomes secreted by cells occur, including changes in RNA, lipids, and proteins, or enhancement of their paracrine effects [45]. These changes significantly affect the biological functions of exosomes and can exert functional effects on recipient cells. Exosomes can be targeted through various mechanisms, including direct binding to plasma membranes, internalization through the endocytosis pathway, or receptor–ligand binding mechanisms. A wide range of cell types, such as cardiomyocytes, pericytes, and infiltrating or resident macrophages, have the ability to both produce and take up exosomes [46]. As a result, exosomes facilitating cross-talk between cardiomyocytes and macrophages may contribute to Dex-mediated regulation of autophagy.

To investigate the impact of Dex-Exos on autophagy regulation, we successfully established a CLP rat model. We then analysed plasma exosomes extracted from rats in sham, CLP, and Dex groups. Notably, Dex-Exos enhanced autophagy and reduced apoptosis in cardiomyocytes. To further investigate the mechanism through which Dex-Exos modulate autophagy, we isolated exosomes from the plasma of rats in the sham, CLP, and Dex treatment groups to characterize the exosomal miRNA profiles of each group. By utilizing miRNA sequencing data, we identified a crucial miRNA element that contributes to our understanding of how Dex influences exosomes. In our study, we identified differentially expressed miRNAs in circulating Exos after Dex intervention. Intriguingly, miR-29b-3p was downregulated in the CLP-Exos group compared to the Sham-Exos group, whereas it was upregulated in the Dex-Exos group compared to the CLP-Exos group. Previous studies have established that miR-29b-3p is closely related to heart disease. miR-29b has been reported to be a positive regulator of cardiac disease. Downregulation of miR-29b-3p is implicated in the pathological processes of heart failure [47]. Forkhead Box O3, pentraxin 3 and NOTCH2 have been reported to be targets of miR-29b-3p [47–49]. However, how miR-29b-3p is involved in the regulation of autophagy and its role in septic cardiac injury remain poorly explored. We further verified by RT-PCR that the plasma level of miR-29b-3p in exosomes in sepsis patients was lower than that in non-sepsis patients.

Given that miRNAs exert their effects by regulating downstream genes, we conducted a screening process to identify potential target genes of miR-29b-3p. This involved the use of gene sequencing and bioinformatics analysis. By screening miRNA target genes via prediction software, we identified GSK-3β as a candidate target gene with seed-matching sites for miR-29b-3p in the 3′-UTR of H9c2 cells. GSK-3β is a serine/threonine kinase involved in the regulation of multiple cellular functions, including glucose metabolism, cell differentiation, proliferation, survival, and apoptosis [26]. Previous studies have demonstrated that GSK-3 can inhibit autophagy through its regulation of mTOR and lysosomes. Overexpression of both GSK-3β and GSK-3α activates mTOR, thus leading to autophagy inhibition by downregulating Beclin-1 and upregulating p62 levels [50]. Our results showed that miR-29b-3p overexpression significantly reduced GSK-3β levels in H9c2 cells and AC16 cells, thus indicating the translational repression of GSK-3β by miR-29b-3p. Moreover, knockdown of GSK-3β in cardiomyocytes resulted in upregulation of the protein expression of the autophagy markers LC3-II/I.

Additionally, when cardiomyocytes were treated with plasma-derived exosomes from rats, we observed decreased GSK-3β Ser9 phosphorylation (indicative of reduced GSK-3β activity) in the CLP-Exos group, whereas it was markedly greater after Dex-exos treatment than in the Sham-Exos group. This finding suggested that the miR-29b-3p/GSK-3β pathway may play a crucial role in regulating autophagy and apoptosis in cardiomyocytes. An understanding of this mechanism is essential for clarifying how Dex treats myocardial injury in sepsis. We aim to further explore whether miR-29b-3p/GSK-3β could serve as a predictor of sepsis progression or a therapeutic target for sepsis.

## Conclusions

In summary, our findings indicate that Dex upregulates miR-29b-3p in macrophage-derived exosomes. This miRNA correspondingly targets the GSK-3β pathway in cardiomyocytes, thus ultimately enhancing the autophagy response. Our experimental results may indicate a new strategy for treating septic myocardial injury.

## Supplementary Material

Fig_S1_final_tkae042

Fig_S2_final_tkae042

final_Supplementary_materials_tkae042

## Data Availability

The authors declare that all data supporting the findings of this study are available within the article.

## Abbreviations

AC16: Human ventricular cardiomyocyte; Alix: Alg-2-interacting protein x; Atg4b: Autophagy-related 4b; CD63: Cluster of differentiation antigen 63; CLP: Cecal ligation and puncture; Dex: Dexmedetomidine; Exos: Exosomes; GSK-3β: Glycogen synthase kinase 3β; H9c2: Embryonic rat cardiomyocyte; ICU: Intensive care unit; LC3: Microtubule-associated-protein light-chain-3; LPS: Lipopolysaccharides; miRNA: MicroRNA; NC: Negative control; NTA: Nanoparticle tracking analysis; PBS: Phosphate-buffered saline; qRT-PCR: Quantitative real-time polymerase chain reaction; siRNA: Small interfering RNA; THP-1: human monocytic cell line; ATCC: American type culture collection; BNCC: BeNa culture collection; mTOR: Mammalian target of rapamycin; ANOVA: Analysis of variance; DMEM: Dulbecco's modified eagle medium; BCA: Bicinchoninic acid; FITC: Fluorescein isothiocyanate; GFP: Green fluorescent proteins; mRFP: Monomeric red fluorescent protein; SDS-PAGE: Sodium dodecyl sulfate polyacrylamide gel electrophoresis; NOTCH2: Notch receptor 2.
