# Supplementary figures and images for: Dexmedetomidine regulates exosomal miR-29b-3p from macrophages and alleviates septic myocardial injury by promoting autophagy in cardiomyocytes via targeting glycogen synthase kinase 3β
Source: Burns Trauma. 2024 Nov 4;12:tkae042. doi: 10.1093/burnst/tkae042 (PMC11534962; doi:10.1093/burnst/tkae042)

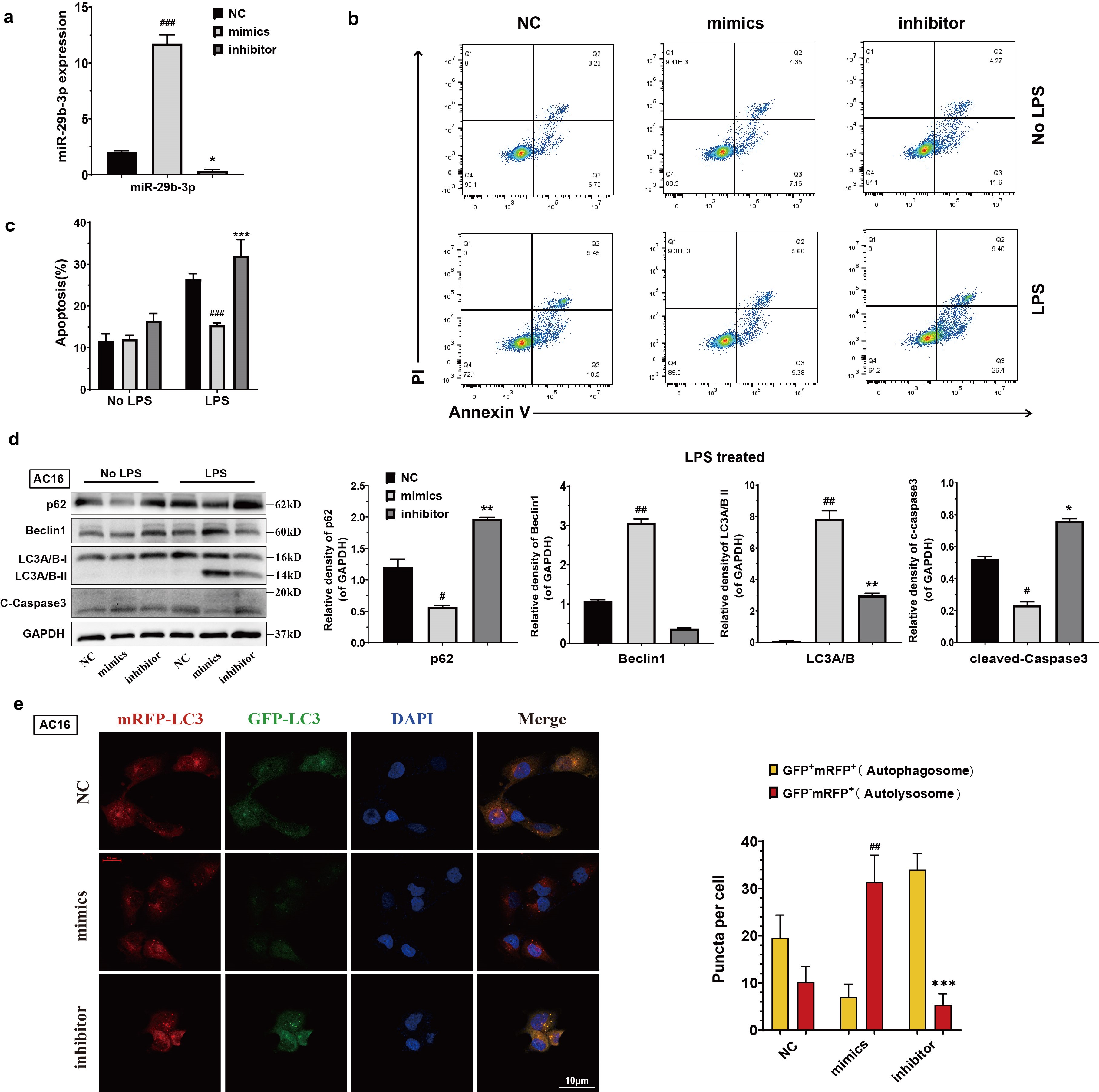

Supplement: Fig_S1_final_tkae042 [file fig_s1_final_tkae042.jpeg]

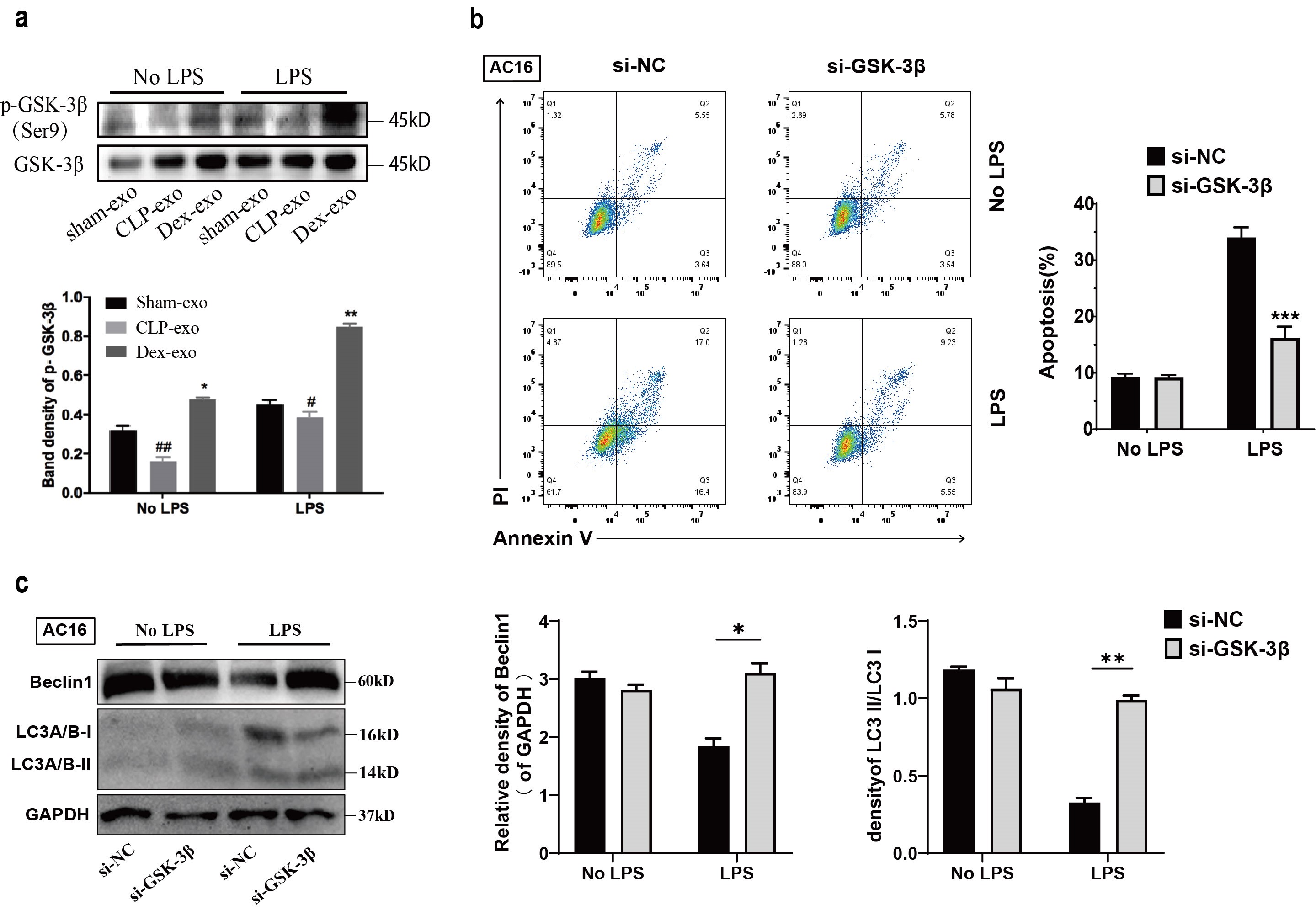

Supplement: Fig_S2_final_tkae042 [file fig_s2_final_tkae042.jpeg]
