# Supplementary material for: Dexmedetomidine regulates exosomal miR-29b-3p from macrophages and alleviates septic myocardial injury by promoting autophagy in cardiomyocytes via targeting glycogen synthase kinase 3β
Source: Burns Trauma. 2024 Nov 4;12:tkae042. doi: 10.1093/burnst/tkae042 (PMC11534962; doi:10.1093/burnst/tkae042)
Supplement: final_Supplementary_materials_tkae042 [file final_supplementary_materials_tkae042.docx]

Supplementary Information

**Figures**

**
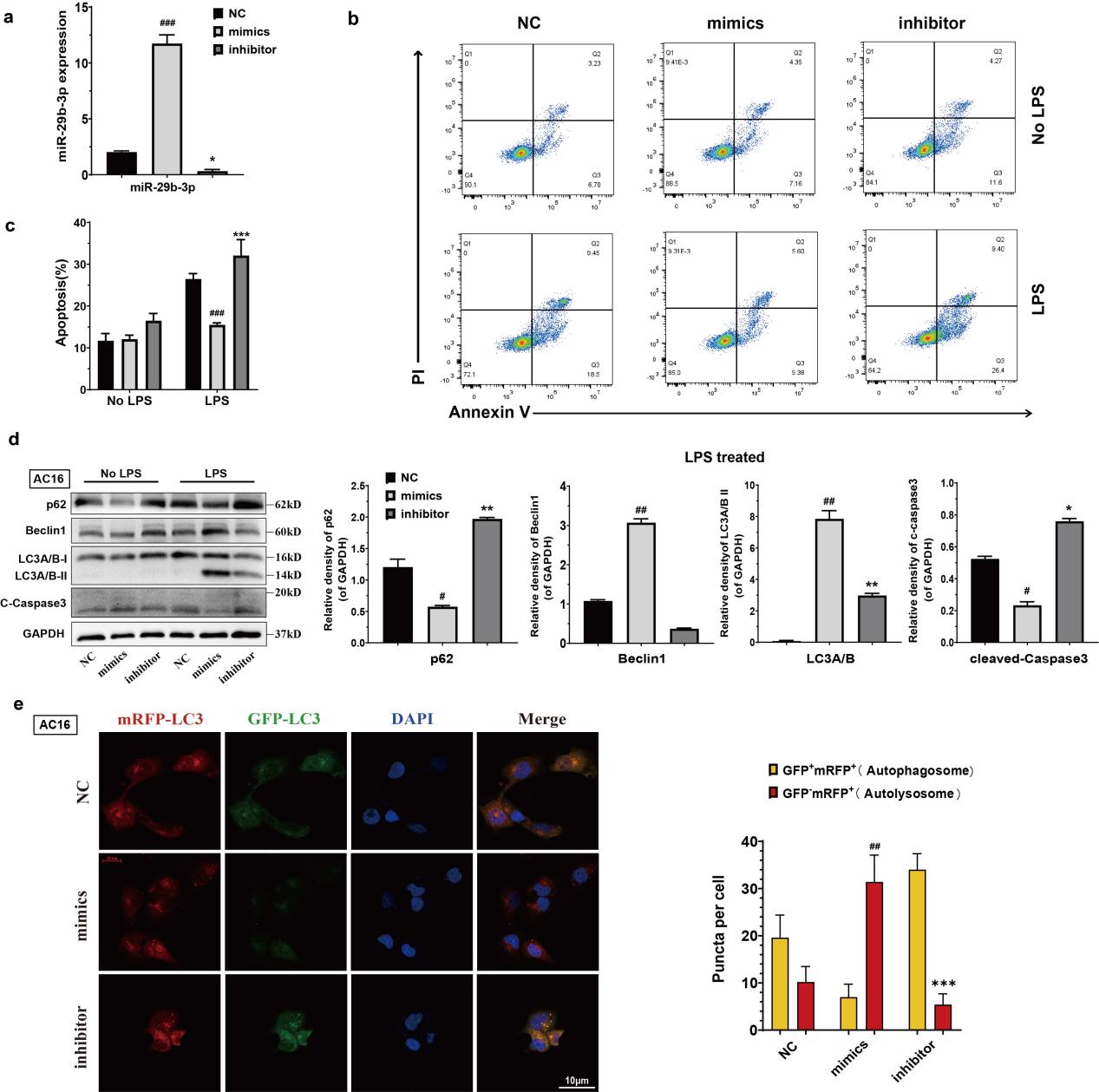
**

**Supplementary Figure S1.** Overexpression of miR-29b-3p promoted autophagy and reduced LPS-induced apoptosis in AC16 cells. **a.** miR-29b-3p expression of AC16 cells transfected with miR-29b-3p mimics, inhibitor, and NC by qRT-PCR detection (n=3, from three independent experiments). **b-c.** miR-29b-3p mimics, inhibitor and NC expressed AC16 cells were incubated without (upper panel) or with LPS (down panel) for 24 hours, and cells were stained with Alexa Fluor647-labeled AnnexinV and PI to determined cellular apoptosis by flow cytometry. **d.** Western blot was used to measure autophagy-related protein (p62, Beclin1 and LC3A/B) and apoptosis-related protein (Cleaved-Caspase3) expression in AC16 cells after up- and down-regulation of miR-29b-3p. **e.** Autophagic flux in each group of AC16 cells. Scale bar=20μm. ^#^P<0.05,^##^P<0.01,^###^P<0.005 vs. mimics group; *P < 0.05,**P < 0.01,***P < 0.005 vs. inhibitor group. *NC* negative control, *GAPDH* glyceraldehyde-3-phosphate dehydrogenase, *PI* propidium iodide, *LPS* lipopolysaccharides, *P62* sequestosome 1, *LC3A/B* microtubule-associated-proteinlight-chain-3 A/B, *C-Caspase3* cleaved cysteine–aspartic acid protease 3, *DAPI* 4',6-diamidino-2-phenylindole.


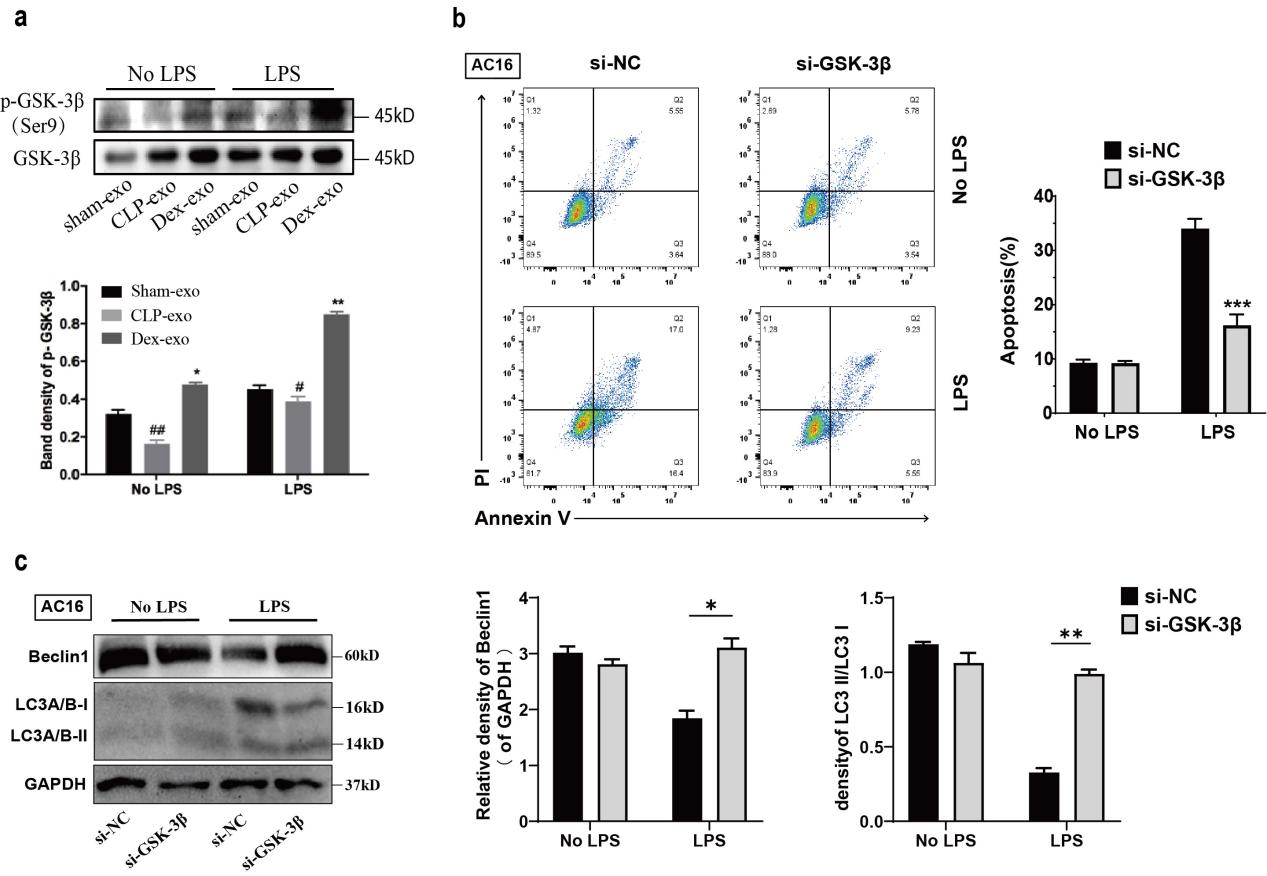


**Supplementary Figure S2.** Down-regulated GSK-3β inhibits apoptosis and promotes autophagy of cardiomyocytes. **a.** Western blot was used to measure phosphorylation level of GSK-3β Ser9 in H9c2 cells after treated with plasma exosomes. **b.** AC16 cells under siRNA transfection were incubated without (upper panel) or with LPS (down panel) for 24 hours, and cells were stained with Alexa Fluor647-labeled Annexin V and PI to determined cellular apoptosis by flow cytometry. **c.** Western blotting for Beclin1 and LC3A/B protein levels in AC16 cells under siRNA transfection. ^#^P<0.05,^##^P<0.01 vs. CLP-exo group; *P < 0.05,**P < 0.01,***P < 0.005 vs. Dex-exo group or si-GSK-3β group. *GSK-3β* glycogen synthase kinase 3β, *CLP* cecal ligation and puncture, *Dex* dexmedetomidine, *Exo* exosomes.

**Tables**

**Supplementary Table 1.** **Patients demographics and clinical parameters.**

Enrolled subjects were characterized as sepsis by a group of blinded critical care physicians(1), or were age- and sex-matched non-sepsis controls subjects. A subset of randomly selected subjects from the ICU Registry with the above diagnoses was selected for analysis. Anonymized plasma samples were generated from blood collected in EDTA-containing tubes obtained from patients within 72 hours of ICU admission and stored at –80°C.


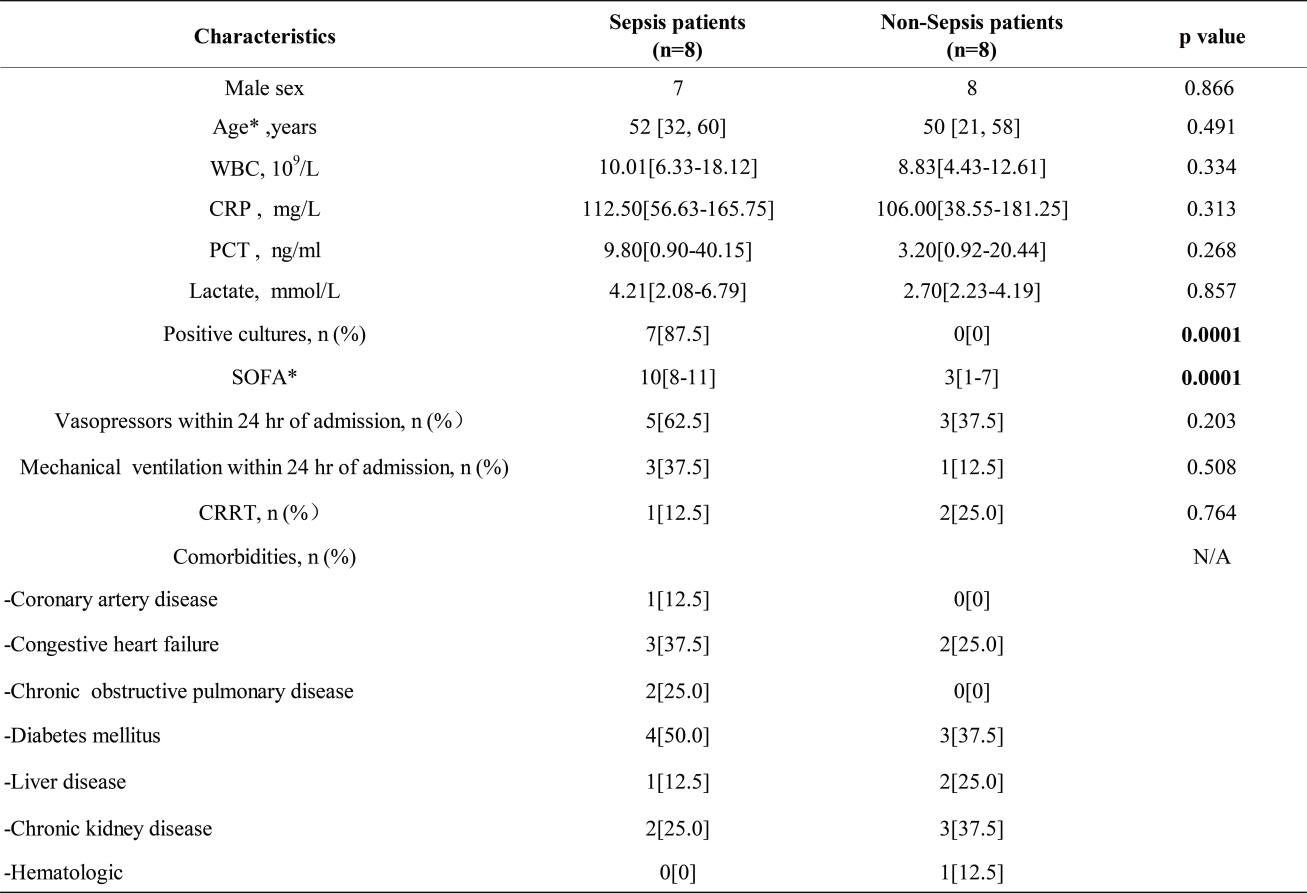


*Age and SOFA scores are expressed in medians [min, max 95% CI].

All other categorical variables are expressed as n (%), and continuous variables were expressed as median [interquartile range]. Chi-square or Fisher exact tests were performed for qualitative variables and MannWhitney U for quantitative variables. SOFA score was measured after 24 hours of ICU stay. Boldface values indicate significance at p< 0.05.

Abbreviations: *EDTA* ethylene diamine tetraacetic acid, *ICU* intensive care unit, *PCT* procalcitonin, *WBC* white blood cell, *CRP* C-reactive protein, *SOFA* sequential organ failure assessment, *CRRT* continuous renal replacement therapy, *N/A* not applicable.

| **Species** | **Target gene** | **Sequence (5’ to 3’)** | |
| --- | --- | --- | --- |
| *Homo sapiens* | *miR-29b-3p* | Forward | CGCGTAGCACCATTTGAAATC |
|  |  | Reverse | AGTGCAGGGTCCGAGGTATT |
|  | *U6* | Forward | AGAGAAGATTAGCATGGCCCCTG |
|  |  | Reverse | ATCCAGTGCAGGGTCCGAGG |
|  | *U6* *stem loop primer* | GTCGTATCCAGTGCAGGGTCCGAGGTATTCGCACTGGATACGACAAAATATG | |
|  | *miR-29b-3p*  *stem loop primer* | GTCGTATCCAGTGCAGGGTCCGAGGTATTCGCACTGGATACGACAACACT | |
| *Rattus norvegicus* | *miR-29b-3p* | Forward | TTCCTATGCATATACTTCT |
|  |  | Reverse | CGTATCCAGTGCGAATA |
|  | *U6* | Forward | ATGACGTCTGCCTTGGAGAAC |
|  |  | Reverse | TCAGTGTGCTACGGAGTTCAG |
|  | *β-actin* | Forward | AAGTCCCTCACCCTCCCAAAAG |
|  |  | Reverse | AAGCAATGCTGTCACCTTCCC |
|  | *Atg4b* | Forward | TGATACTCTCCGGTTTGCCG |
|  |  | Reverse | TCATCTGTCCACACCGAAGC |
|  | *Bnip3* | Forward | CACTTTGCAGTCCCCCTCTT |
|  |  | Reverse | ACTGCCCACCCAAGGTAATG |
|  | *GSK-3β* | Forward | CGAACTCCACCAGAGGCAAT |
|  |  | Reverse | CAGAAGCGGCGTTATTGGTC |
|  | *U6 stem loop primer* | GTCGTATCCAGTGCAGGGTCCGAGGTATTCGCACTGGATACGACAAAATA | |
|  | *miR-29b-3p*  *stem loop primer* | GTTGGCTCTGGTGCAGGGTCCGAGGTATTCGCACCAGAGCCAACATACAC | |

**Supplementary Table 2.** Primers used for qRT-PCR.

Abbreviations: *qRT-PCR* real-time quantitative reverse transcription polymerase chain reaction, *Atg4b* autophagy-regulating protease 4b, *Bnip3* bcl2 interacting protein 3, *GSK-3β* glycogen synthase kinase 3β.

**Supplementary Table 3.** Antibodies used for western blotting.

| **Antibody** | **Vendor Cat no.** | **Local** | **Country** | **Titer** |
| --- | --- | --- | --- | --- |
| Alix | Santa Cruz Biotechnology  sc-53540 | Dallas, TX | USA | 1:500 |
| CD63 | Santa Cruz Biotechnology  sc-5275 | Dallas, TX | USA | 1:500 |
| β-actin | Proteintech  66009-1-Ig | Shanghai | China | 1:5000 |
| Beclin1 | Cell signaling Technology  3495S | Shanghai | China | 1:1000 |
| LC3A/B | Cell signaling Technology  12741S | Shanghai | China | 1:1000 |
| P62 | Cell signaling Technology  5114S | Shanghai | China | 1:1000 |
| Cleaved-Caspase3 | Cell signaling Technology  9661S | Shanghai | China | 1:1000 |
| GAPDH | Cell signaling Technology  5174S | Shanghai | China | 1:1000 |
| GSK-3β | Cell signaling Technology  9315S | Shanghai | China | 1:1000 |
| Phospho-GSK-3β (Ser9) | Abcam  ab131097 | Shanghai | China | 1:1000 |
| Phospho-GSK-3β(Ser9) | Invitrogen  MA5-14873 | Shanghai | China | 1:500 |
| α/β-Tubulin | Cell signaling Technology  2148S | Shanghai | China | 1:1000 |

# Abbreviation: *Alix* alg-2-interacting protein x, *CD63* cluster of differentiation antigen 63, *Cleaved-Caspase3* cleaved cysteine–aspartic acid protease 3, *LC3A/B* microtubule-associated-proteinlight-chain-3 A/B, *P62* sequestosome 1, *GAPDH* glyceraldehyde-3-phosphate dehydrogenase, *GSK-3β* glycogen synthase kinase 3β.

**Supplementary Table 4.** Reagents used for cell treating.

| **Reagent** | **Cat.no** | **Company** |
| --- | --- | --- |
| LY294002 | S1105 | Selleck |
| SB216763 | S1075 | Selleck |
| PMA | P1585 | Millipore Sigma |
| LPS | L4391 | Millipore Sigma |
| Dexmedetomidine hydrochloride | SML0956 | Millipore Sigma |

Abbreviation: *PMA* phorbol 12-myristate 13-acetate, *LPS* lipopolysaccharides.

**References**

(1) Singer M, Deutschman CS, Seymour CW, Hari [M](https://webvpn.shsmu.edu.cn/https/77726476706e69737468656265737421e0e243912234265e7d0a80e296592e7bb7d62ae2c192eb/?term=Shankar-Hari+M&cauthor_id=26903338), Annane D, Bauer [M](https://webvpn.shsmu.edu.cn/https/77726476706e69737468656265737421e0e243912234265e7d0a80e296592e7bb7d62ae2c192eb/?term=Bauer+M&cauthor_id=26903338), et al. The Third International Consensus Definitions for Sepsis and Septic Shock (Sepsis-3). JAMA. 2016;315(8):801-810.
